# Supplementary material for: A Global Screen for Assembly State Changes of the Mitotic Proteome by SEC-SWATH-MS
Source: Cell Syst. 2020 Feb 26;10(2):133–155.e6. doi: 10.1016/j.cels.2020.01.001 (PMC7042714; doi:10.1016/j.cels.2020.01.001)

Q9Y4D8 | HECD4_HUMAN | HECTD4 C12orf51 KIAA0614
Monomer MW [kDa]: 439.344 Monomer expected elution fraction: 27

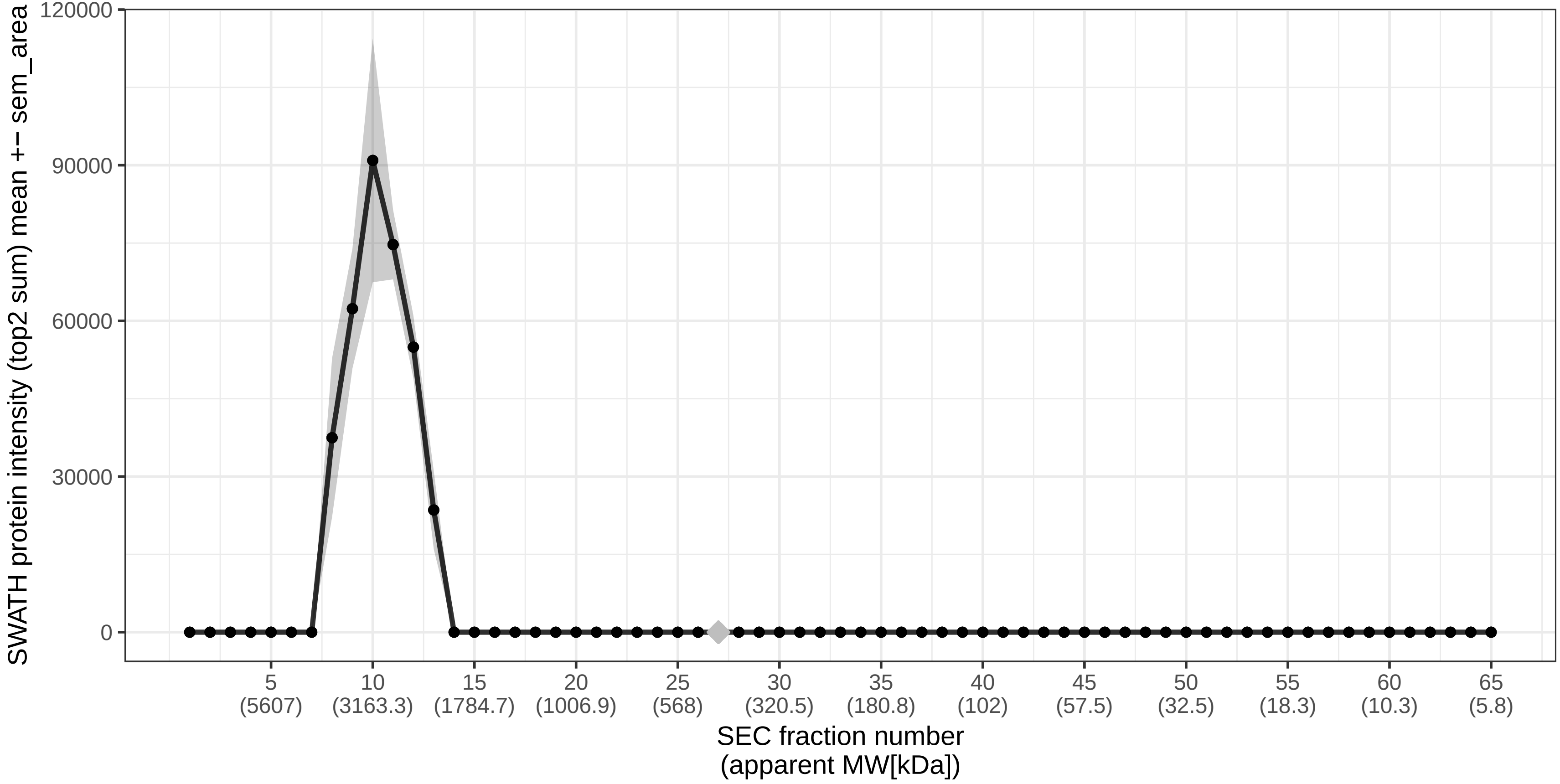

Supplement: Data S1. SEC-SWATH-MS Protein Chromatograms, Related to Figure 1 [file mmc6.zip › SECchrom_Q9Y4D8_HECD4_HUMAN_HECTD4_C12orf51_KIAA0614.pdf]
